# Imaging diagnosis and treatment selection for brain tumors in the era of molecular therapeutics

**DOI:** 10.1186/s40644-022-00455-5

**Published:** 2022-04-18

**Authors:** Saivenkat Vagvala, Jeffrey P. Guenette, Camilo Jaimes, Raymond Y. Huang

**Affiliations:** 1 Division of Neuroradiology, Brigham and Women’s Hospital, Dana-Farber Cancer Institute, 75 Francis St, Boston, MA 02115 USA; 2 Division of Neuroradiology, Boston Children’s, 300 Longwood Ave., 2nd floor, Main Building, Boston, MA 02115 USA

**Keywords:** IDH, Medulloblastoma, BRAF, 1p19q, EGFR, H3 K27-altered, FGFR3-TACC3, TERT, Glioma

## Abstract

Currently, most CNS tumors require tissue sampling to discern their molecular/genomic landscape. However, growing research has shown the powerful role imaging can play in non-invasively and accurately detecting the molecular signature of these tumors. The overarching theme of this review article is to provide neuroradiologists and neurooncologists with a framework of several important molecular markers, their associated imaging features and the accuracy of those features. A particular emphasis is placed on those tumors and mutations that have specific or promising imaging correlates as well as their respective therapeutic potentials.

## Background/introduction

In the era of molecular analysis of neural axis tumors, there is a greater impetus to non-invasively predict molecular markers to guide therapy and prognostication. Imaging technologies have become essential in this regard. Imaging allows qualitative assessment of tumor burden and extent prior to and following local and systemic therapy. Additionally, it is increasingly utilized as both a qualitative and quantitative biomarker to differentiate tumor types.

The current standard of care imaging relies heavily on conventional MRI with the workhorse FLAIR/T2 and T1-weighted sequences. More advanced MR techniques, including perfusion and diffusion weighted imaging as well as spectroscopy are being increasingly utilized in clinical and research capacities to help predict tumor types. PET imaging with amino acid tracers has been of interest as the physiologic information it provides complements the structural information afforded by MRI.

The current gold standard of tissue sampling is accompanied not only by obvious risks and complications, but by suboptimal sampling as many gliomas can be heterogenous. This in turn may not accurately reflect the tumoral phenotype in its entirety and can potentially miss critical genomic aberrations. The nascent field of radiomics/radiogenomics/artificial intelligence (AI) is of great interest as it can help predict tumoral genotype and/or provide higher yielding targetable biopsy sites within heterogenous tumors. Additionally, the field may prove useful with patient counseling where a conservative approach may be employed if the MR imaging features suggest a low-grade glioma with favorable genomic signature [1]. Other anticipated clinical roles include reclassifying tumors previously diagnosed before the 2016 WHO update, guiding perioperative management and post-treatment follow-up, as well as predicting non-canonical IDH mutations [1].

The goal of this review article is to provide neuroradiologists and neurooncologists with an outline of currently known important molecular markers, their associated imaging features, and the accuracy of those features. The selection of CNS tumors described is based on our survey of the current literature for those tumor types that carry targetable mutations with recently completed or ongoing clinical trials. We will emphasize the anticipated role of imaging in patient selection for treatment regimens of several brain tumors including diffuse glioma, medulloblastoma and BRAF-mutant tumors.

## Diffuse glioma

### Background

Traditionally, tumor histology dominated classification and grading schema. However, 2016 and newly released 2021 updates to the World Health Organization (WHO) classification of central nervous system (CNS) tumors have integrated molecular parameters and in certain instances has emphasized them above histology [2, 3]. The most notable changes involved diffuse infiltrative gliomas with regards to glioblastoma classification, isocitrate dehydrogenase (IDH) and 1p19q codeletion statuses.

**Fig. 1:**
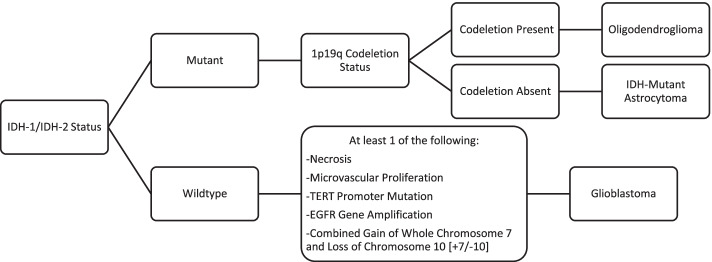
Simplified diagram outlining the general diagnostic tree with regards to adult-type diffuse gliomas

Previously, tumors harboring an IDH mutation in the absence of a 1p19q codeletion were classified as diffuse or anaplastic astrocytoma and secondary glioblastoma. Those tumors now have a unifying diagnosis of astrocytoma. In the setting of both an IDH mutation and a 1p19q codeletion, the diagnosis is oligodendroglioma [3]. Glioblastoma on the other hand is now considered a separate entity and must be IDH-wildtype (Fig. 1) [3].

The importance of predicting these molecular statuses has prompted considerable research into imaging correlates for IDH and 1p19q statuses. Additional molecular biomarkers of interest due to their targetable potentials include epidermal growth factor receptor (EGFR) amplification and mutation, histone H3F3A gene (H3 K27-altered), fibroblast growth factor receptor 3-transforming acidic coiled-coil containing protein (FGFR3-TACC3) fusions and telomerase reverse transcriptase (TERT) promoter mutations [4].

As a testament to the growing reliance on molecular status, if there is discordance between a tumor’s histologic phenotype and genotype, it is the genotype that determines diagnosis and treatment [3]. For example, if an adult IDH-wildtype diffuse astrocytic tumor shows low-grade histologic features yet harbors one or more of 3 key genetic alterations (TERT promoter mutation, EGFR gene amplification and/or combined gain of entire chromosome 7 and loss of entire chromosome 10 [+ 7/-10]), it is considered glioblastoma [3].

A salient point that the WHO 2021 update emphasizes is the separation of the prognostically and biologically distinct groups of adult-type and pediatric-type diffuse gliomas [3]. Some genetic markers are unique to each group. For instance, FGFR3-TACC3 fusion and TERT promoter mutation are more closely associated with adult-type glioma, H3 K27-altered mutation is associated with pediatric-type glioma and EGFR mutation to both.

### IDH mutant clinical implications

Gliomas with IDH mutation confer better prognoses (median survival of ~ 31 months), whereas those that are IDH-wildtype have a poor prognosis (median survival of ~ 15 months) [5]. As IDH-mutant gliomas have a more favorable survival, a less aggressive treatment approach may be utilized [6]. Additionally, there are ongoing efforts to develop IDH enzyme inhibitors to enhance canonical therapies [7].

The prediction of 1p19q codeletion has implications on treatment regimens. Patients with anaplastic oligodendrogliomas are commonly treated with radiation and temozolomide as modeled after the standard treatment for glioblastoma. However, analyses of patients carrying the codeletion showed that treatment with radiation plus a PCV (procarbazine, CCNU/lomustine and vinscristine) chemotherapy regimen resulted in a significant improvement in survival curves after ~ 7 years compared to treatment with radiation alone [8, 9]. Currently, the comparative efficacy of radiation with temozolomide versus radiation with PCV remains elusive but will be addressed by the ongoing CODEL study [10].

### IDH mutant imaging

While conventional MRI sequences can help distinguish low-grade from high-grade gliomas by assessing for edema, enhancement, hemorrhage, necrosis, multifocality and/or multicentricity, there remains overlap in their appearances [11]. In turn, several specific imaging signs for molecular prediction of gliomas have surfaced.

**Fig. 2:**
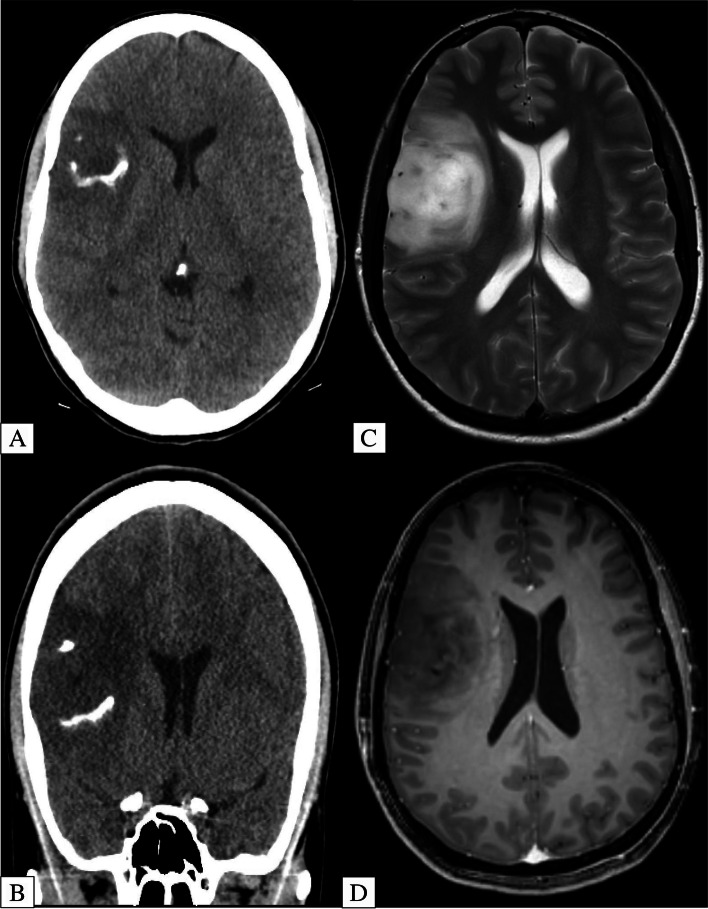
**A-D** 38-Year-Old Patient with Biopsy Proven Right Frontal Lobe Oligodendroglioma. Axial and coronal unenhanced CT (**A**/**B**) show a hypodense right frontal lobe lesion with gyriform/ribbon calcification. Axial T2-Weighted MRI (**C**) shows a corroborative T2 hyperintense lesion with no substantial enhancement on axial T1-Weighted Post Contrast MRI (**D**)

For example, imaging features suggestive of oligodendrogliomas (IDH-mutant, 1p19q codeleted) include poorly defined, heterogenous mass with calcifications (Fig. 2) [12–14]. A recent systematic review by Lasocki et al. summarized that frontal lobe location is suggestive of an IDH mutation with oligodendroglioma slightly favored over IDH-mutant astrocytoma. A temporal lobe location is unlikely to be oligodendroglioma [15]. Diffusion weighted imaging (DWI) can help differentiate oligodendrogliomas from astrocytomas as the former tend to have lower apparent diffusion coefficient (ADC) values [13, 16].

**Fig. 3:**
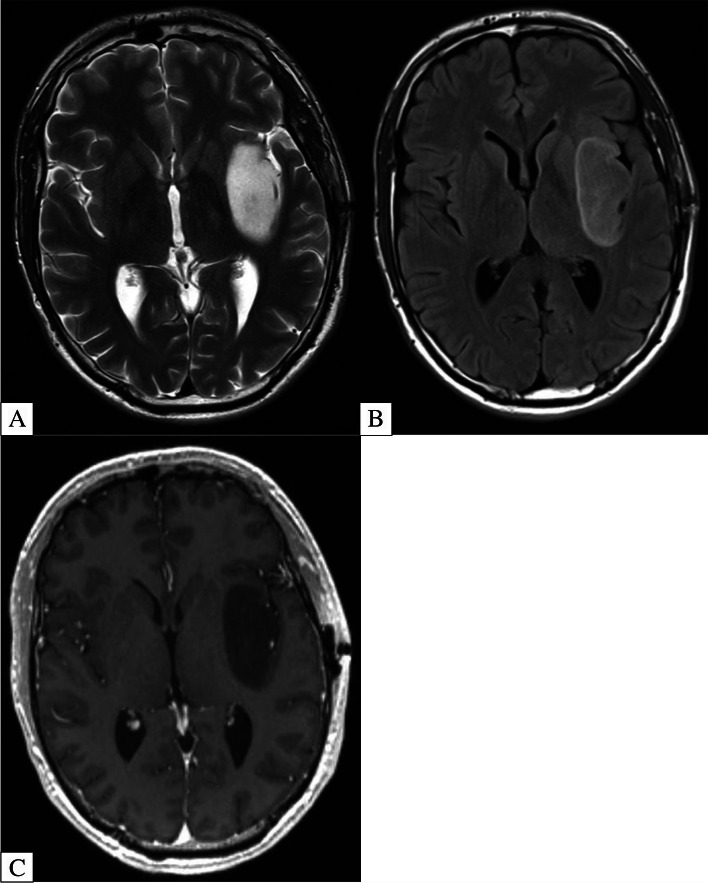
**A-C** 26-Year-Old Patient with Biopsy Proven IDH-Mutant Astrocytoma Showing the T2/FLAIR Mismatch Sign. Axial T2-Weighted MRI (**A**) shows a homogenously T2 hyperintense lesion centered within the left insula. Axial FLAIR MRI (**B**) shows the lesion becomes relatively hypointense with peripheral hyperintense rim due to incomplete suppression. Axial T1-Weighted Post Contrast MRI (**C**) shows no substantial enhancement within the lesion

A highly specific, yet insensitive imaging signature for diffuse astrocytoma (IDH-mutant, 1p19q non-codeleted) is the T2/FLAIR mismatch sign (Fig. 3) [17–19]. The specificity of this sign has been validated in multiple studies and is currently gaining traction in clinical practice. In fact, a 2021 meta-analysis showed a pooled specificity and sensitivity of 100% and 42%, respectively [20]. It is important to note that the strikingly perfect specificity of this sign is contingent upon stringent adherence to imaging criteria including (i) complete or near-complete, homogenous signal of the tumor on T2-weighted sequence with (ii) hypointense signal on FLAIR sequence except for a hyperintense peripheral rim [21]. Typically there should be minimal to no associated enhancement and this sign should not be applied to pediatric patients [21].

PET imaging utilizing amino acid tracers such as methyl-11C-L-methionine (MET), 3,4-dihydroxy-6-[18F]-fluoro-L-phenylalanine (FDOPA) and 18F-fluoroethyltyrosine (FET) has shown promising influence on treatment of brain tumors. For example, higher-grade gliomas could be classified with a sensitivity and specificity of > 80% by utilizing dynamic FET/PET imaging where they exhibit more rapid uptake and washout compared with lower-grade gliomas [22, 23]. FDOPA/PET showed an accuracy of 82% for distinguishing true from pseudo-progression in patients with glioblastoma [24]. Unfortunately, the ability to predict the IDH and 1p19q codeletion statuses of gliomas by PET has been challenging. For instance, 1p19q co-deletion status has been associated with both lower and higher MET uptake on a series of studies [25–27].

MR spectroscopy is a quantitative tool that can further differentiate low from high-grade tumors. Higher grade tumors exhibit lower N-acetylaspartate (NAA) and higher choline, which are markers of neuronal viability and cell membrane turnover, respectively. An additional metabolite of interest is 2-hydroxyglutarate (2HG). As a result of the IDH mutation, there is a gain of enzymatic function that generates 2HG within tumor cells and results in DNA hypermethylation [28]. It is not seen in high concentrations in normal brain tissue or IDH-wildtype tumors [29, 30]. Based on meta-analyses, the sensitivity and specificity of 2HG for the presence of IDH mutation is ~ 91% and 95%, respectively [31, 32].

The past decade has been dominated by progress in the field of AI and radiomics. This allows image feature characterization and analyzation to extract information that is difficult or impossible to obtain by human vision. Such data includes texture analysis and diffusion kurtosis or the non-Gaussian movement of tissue water molecules. For instance, IDH mutation prediction using an AI approach for feature extraction have shown a pooled sensitivity and specificity of 87% and 90%, respectively. The overall accuracy for predicting IDH-wildtype vs IDH-mutant/1p19q codeletion vs IDH-mutant/1p19q non-codeletion was 78.2% [33, 34].

### EGFR mutant clinical implications

It has been shown that high EGFR expression correlates with poor prognosis [35]. This makes it a molecular target with therapeutic, diagnostic and prognostic potentials. The clinical impact has yet to be understood as current EGFRvIII targeting therapies have not yet shown a survival benefit, including the anticipated rindopepimut vaccine [35, 36]. However, there is an early promising result with autologous chimeric antigen receptor (CAR) T cells targeted to EGFRvIII. A patient survived 36 months after disease recurrence, which exceeded expected survival for recurrent glioblastoma. Tissue analysis from surgical resection showed long term immunosuppressive adaptive changes in the tumor, reduced EGFRvIII expression and a significant reduction in relative cerebral blood volume (rCBV) following CAR T treatment [37].

### EGFR mutant imaging

**Fig. 4:**
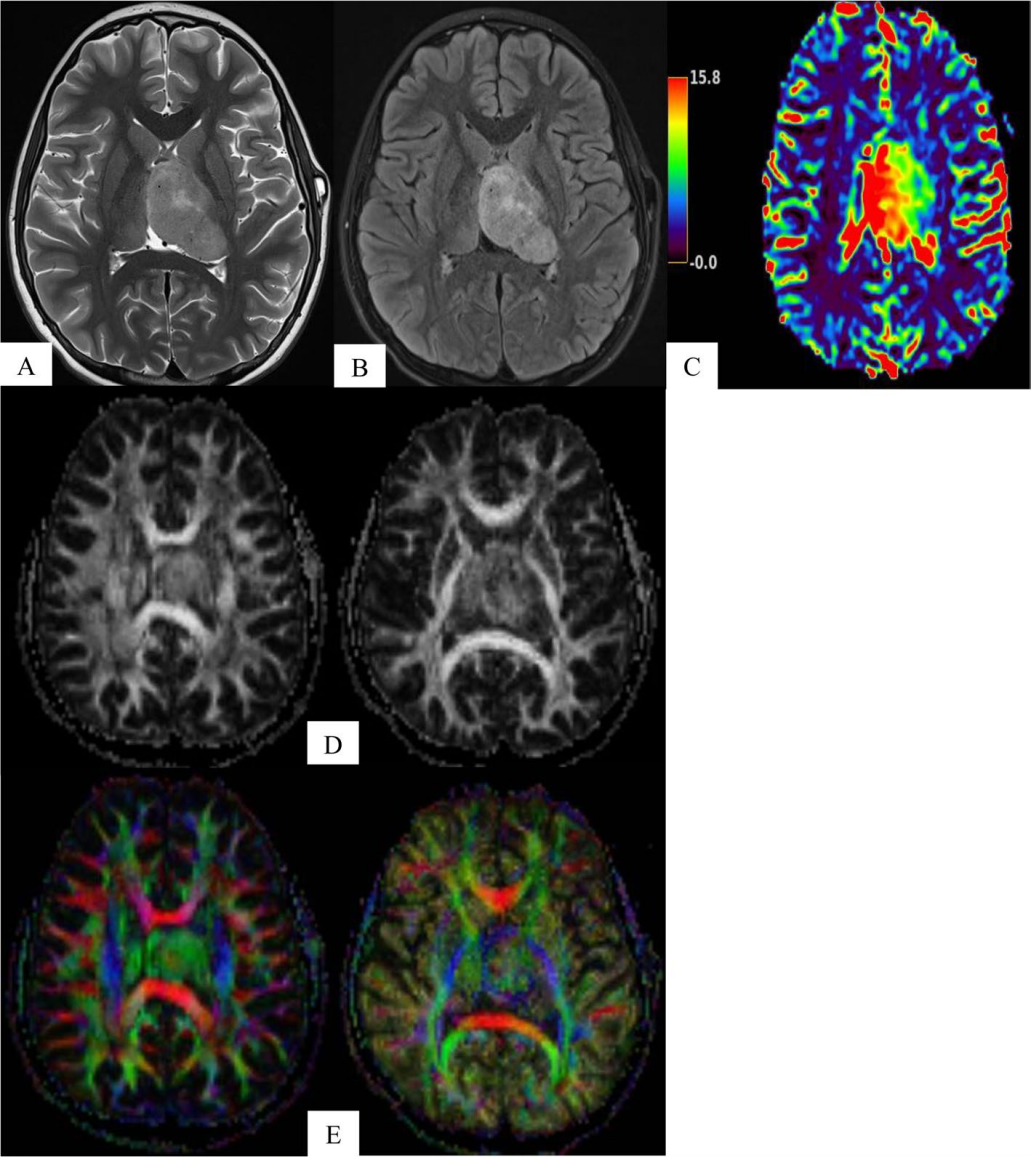
**A-E** 11-Year-Old Patient with Biopsy Proven Left Thalamic High-Grade Glioma with EGFRvIII Mutation. Axial T2-Weighted (**A**) and FLAIR MRI (**B**) show a large left thalamic mass with overall low signal intensity. MRI perfusion (**C**) shows elevated rCBV. Fractional anisotropy (**D**) and corresponding color-coded vector maps (**E**) show high signal

With regards to imaging, the EGFRvIII mutation is of particular interest as it is tumor specific and absent in normal tissues. Sensitive and specific EGFRvIII mutation radiomic signatures in glioblastoma have been identified including higher rCBV, lower ADC, higher fractional anisotropy (FA), lower T2-FLAIR, and a more variable spatial pattern (Fig. 4) [38]. The spatial distribution of the tumor was the most distinctive feature of this mutation. Tumors harboring the mutation typically overlapped the frontoparietal lobes, whereas those negative for the mutation were found predominantly in the temporal lobe [38].

### FGFR3-TACC3 mutant clinical implications

An additional tumorigenic mutation of interest is the FGFR3-TACC3 fusion, which can be seen in up to 3% of gliomas. Di Stefano et al. analyzed the clinical, molecular and radiomic profiles of such gliomas with this mutation and found that it was mutually exclusive with IDH mutation and EGFR amplification [39]. It is also associated with longer survival and better clinical outcomes. Thus FGFR3-TACC3 fusion has become a new therapeutic target. The effects of the specific FGFR inhibitor, JNJ-42756493, was examined in pre-clinical experiments and was shown to inhibit growth of gliomas harboring the FGFR3-TACC3 in vitro and in vivo. In fact, two patients in the Stefano et al. cohort showed clinical improvement and minor treatment response, respectively [40].

### FGFR3-TACC3 mutant imaging

Radiologic features of these gliomas typically manifest as non-eloquent area involvement with poorly defined margins and reduced enhancement intensity [39]. Furthermore, radiomic data of this tumor profile shows good accuracy that has been confirmed on both exploratory and validation cohort, which may be advantageous in predicting this tumor type non-invasively [39].

### TERT promoter mutant clinical implications

Telomerase reverse transcriptase (TERT) is the catalytic subunit of telomerase, an enzyme responsible for defining cells’ lifespan and stability. TERT promoter mutations result in an unlimited proliferative capacity of tumor cells and have been reported in up to 80% of glioblastoma [41]. These mutations are associated with a worse prognosis thus requiring more aggressive treatment [42, 43]. As normal cells have a lower telomerase activity compared to cancer cells, targeted telomerase-inhibitor therapy has become an attractive opportunity for exploration.

Since multiple molecular pathways lead to telomerase activation, there are several targeting strategies including vaccines and immunotherapies [44]. To date, there are no approved TERT promoter glioblastoma therapies. However, there are several supportive in vitro studies and ongoing in vivo trials. For example, eribulin (a microtublublin inhibitor with specific activity against TERT-RNA-dependent RNA polymerase), imetelstat (a TERT inhibitor), and BIBR1532 (a potent telomerase inhibitor) have showed promising results with glioblastoma cell lines [45–47]. A phase I/II trial on seven glioblastoma patients receiving a TERT activity-targeted vaccine showed that all recipients had statistically significant longer progression free survival compared to historical-matched controls (694 days vs 236 days) [44, 48]. Currently, there is an ongoing phase I/II trial on UCPVax, a telomerase-derived vaccine, for treatment of glioblastoma [49].

### TERT promoter mutant imaging

**Fig. 5:**
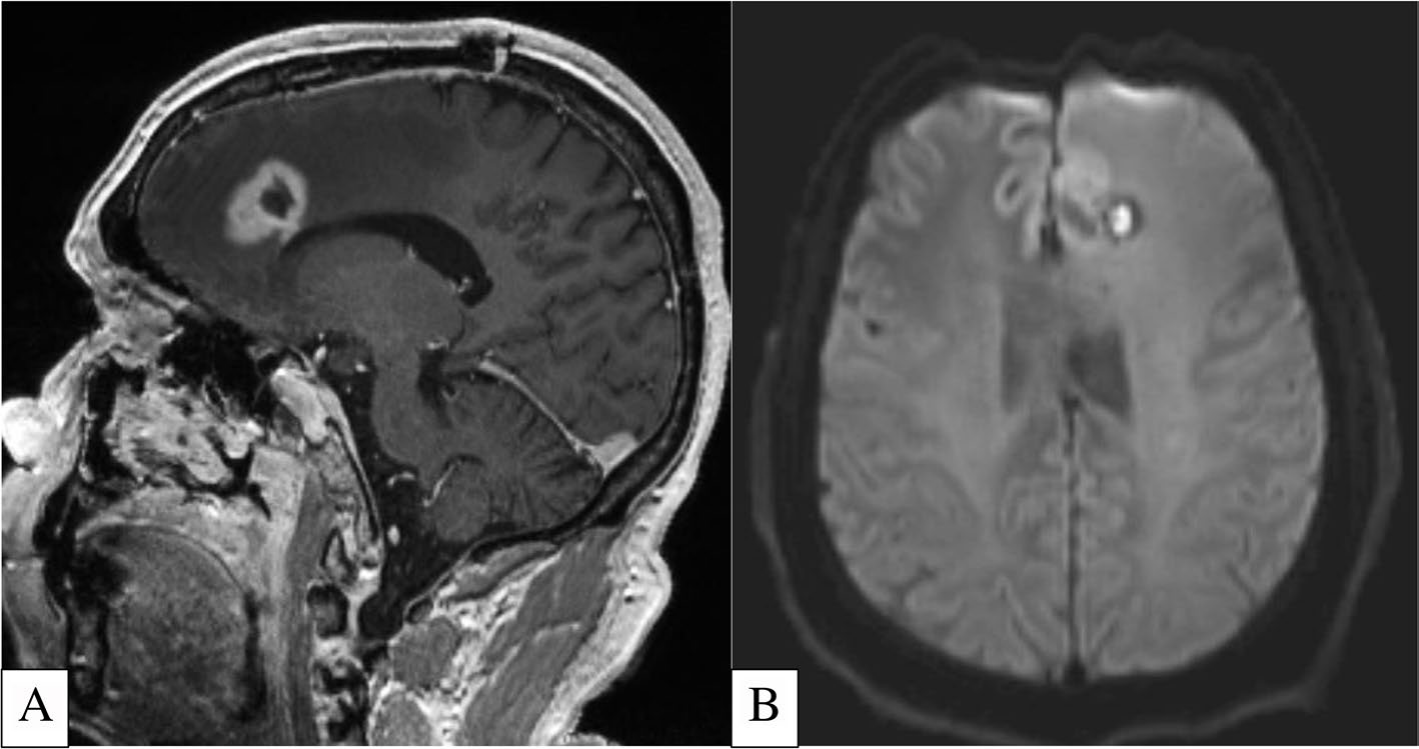
**A-B** 53-Year-Old Patient with Biopsy Proven Left Frontal Lobe Glioma with TERT Promoter Mutation. Sagittal T1-Weighted Post Contrast MRI (**A**) shows an enhancing lesion with central non-enhancing component that exhibits elevated DWI signal (**B**), in keeping with necrosis

A couple studies utilizing AI/radiomics have shown that high grade gliomas with TERT promoter mutation are associated with higher volumes of necrosis (Fig. 5) [50, 51]. In a small sample size, Ivanidze et al. demonstrated that glioblastoma with TERT promoter mutation is associated with lower vascular permeability values (K^trans^ and k_ep_). Their findings also suggested that there is a greater risk of death with increasing blood–brain barrier dysfunction in TERT-mutated but not TERT-wildtype tumors [52]. Additionally, Fukuma et al. were able to successfully classify gliomas with IDH-wildtype, IDH/TERT promoter co-mutation as well as IDH-mutant/TERT-wildtype genomic signatures with a 63.1% accuracy utilizing a pre-trained convolutional neural network [53].

### H3 K27-altered clinical implications

A subset of diffuse glioma that frequently occurs at midline predictably carries the H3 K27-alteration. This mutation predominantly affects children and to a lesser degree adults. While prognosis is consistently poor in the pediatric realm, the prognosis in adults is more variable [54, 55]. Due to the central location of these tumors, surgical diagnosis can be challenging. To improve our understanding of the molecular pathways driving oncogenesis and progression in these aggressive set of tumors, biopsy is being adopted in cases of suspected H3 K27-altered tumors [56, 57].

Currently, standard therapies with radiation and temozolomide have largely failed to improve survival [58]. Thus candidate target drugs including the epigenetic modifier panobinostat and GSKJ4, an inhibitor of the Jumonji-domain demethylase H3K27, are being explored [59, 60].

### H3 K27-altered imaging

The characteristics of tumors with this mutation on standard imaging is variable ranging from mass-like, non-enhancing expansion without necrosis to an aggressive, infiltrative, necrotic and enhancing mass [61]. Lower ADC values at baseline, high skewness on ADC histogram analysis, high volume of enhancing tumor, and rim enhancement are associated with a worse prognosis [62, 63]. While these imaging features are not specific for this mutation, recent advances in radiomics/AI have been promising in predicting this mutational status [64–72]. For example, Jaimes et al. reported that the volume of enhancing tumor and ADC histogram parameters significantly differed between various types of histone mutant tumors (H3F3A, HIST1H3B, HIST1H3C) [73]. Further validation and utilization of these radiomic signatures can help translate into a changing treatment paradigm.

## Medulloblastoma

### Background

Medulloblastoma is the most common malignant brain tumor of childhood. In keeping with the greater emphasis on tumoral molecular status, the WHO classification initially recognized four principle molecular groups of this tumor including wingless type (WNT), sonic hedgehog (SHH), and groups 3 and 4 [74, 75]. The 2021 WHO update further divided SHH on the basis of *TP53* status (wildtype vs mutant) due to vastly different clinicopathologic natures. Additionally, groups 3 and 4 are now designated under non-WNT/non-SHH medulloblastomas and multiple more granular subgroups were added (4 of SHH and 8 of non-WNT/non-SHH) [3].

### Medulloblastoma clinical implications

WNT tumors typically have an excellent prognosis, SHH and group 4 have an intermediate prognosis and group 3 tumors have a relatively poor prognosis (> 90%, ~ 75% and 50–60% survival at 5 years, respectively) [76, 77]. Risk adapted treatment is based on clinical factors including metastatic disease at presentation and residual disease after surgical resection. Given these divergent prognoses, there is considerable capacity to under- or over-treat a subgroup if the molecular landscape is not accounted for.

In patients with a WNT tumor and no metastatic disease, a de-escalated regimen with reduced dose craniospinal radiation and/or chemotherapy can be considered [6]. Patients with SHH subgroup γ can be treated with chemotherapy only whereas those with SHH subgroup β may require intraventricular methotrexate in addition to chemotherapy [78]. Currently, the addition of gemcitabine, a nucleoside analog, and pemetrexed, a folate antimetabolite, are being evaluated in whether they confer an improved prognosis in group 3 and 4 medulloblastoma [79, 80].

### Medulloblastoma imaging

The majority of medulloblastomas arise in the cerebellum. The epicenter of the tumor has been shown to be predictive of the molecular group. WNT tumors typically are centered at the cerebellar peduncle, adult SHH tumors within the cerebellar hemispheres and group 3/4 tumors at midline [74, 75, 81, 82]. Additionally, infants with a tumor that exhibits ill-defined margins and prominent enhancement is likely to be of the group 3 or SHH variety. In contrast, children with a tumor that exhibits well-defined margins, but trace to no enhancement is likely to be group 4. Medulloblastomas found in adulthood tend to be of the SHH variety [74].

MR spectroscopy can help differentiate groups 3 and 4 as these show taurine peaks and high creatine. On the other hand, the SHH group shows low to no taurine or creatine levels [83, 84].

Within the past few years, there has been growing literature on utilizing radiomics and machine learning to predict these molecular groups. Dasgupta et al. have shown a model in which medulloblastoma groups could be accurately predicted in 74% cases, the most impressive of which was the SHH group at 95% [85]. This degree of accuracy has been corroborated on multiple additional analyses [86–88].

The pattern of tumor dissemination in metastatic medulloblastoma also demonstrates unique radiomic signatures [89]. Metastases of group 3 tumors have a laminar appearance, metastases of group 4 tumors are nodular, and suprasellar metastatic deposits are highly specific of group 4 tumors [90].

## BRAF mutant tumors

### Background

Oncogenesis in pediatric gliomas differs significantly from adult tumors. In children, most tumors of glial origin are low-grade. From a molecular perspective, virtually all of them carry mutations that affect the mitogen activated protein kinase (MAPK) pathway [91]. The canonical mutations that drive oncogenesis and carry prognostic significance in adult gliomas (e.g. IDH) are not involved in oncogenesis in low-grade tumors of childhood. These biologic differences are believed to drive the diverging clinical course of low-grade gliomas in children and adults [92].

BRAF is a protooncogene that is part of the MAPK pathway. It is a commonly implicated mutation in several pediatric brain tumors, particularly low-grade gliomas. The two principal alterations are BRAF fusion and the V600E mutant [93].

The chromosomal fusion alteration involves duplication and insertion of the BRAF oncogene into a fusion target, the most common of which is the K1AA1549 gene [93]. The BRAF-K1AA1549 fusion has been reported in up to 66% of pilocytic astrocytoma (PA) [94]. The evidence for specificity of BRAF status in PA remains controversial. Some reports suggest no cases of BRAF fusion in a range of low-grade gliomas, whereas other cohorts report the alteration in up to 15% of non-pilocytic low-grade gliomas [95]. *BRAF*^V600^ mutation has been implicated in high frequencies with pleomorphic xanthoastrocytoma (PXA), ganglioglioma (GG) and extracerebellar PA [96].

### BRAF mutant tumor clinical implication

Harboring the BRAF-K1AA1549 fusion is an independent prognostic marker for significantly improved 5-year progression free survival [97]. By confirming BRAF fusion status, the therapeutic milieu is expanded to include novel BRAF mitogen-activated protein kinase kinase (MEK) inhibitors such as U0126, PD0325901 and AZD6244. These act by blocking proliferation and arresting growth of glioma cells [98–100].

While *BRAF*^V600^ mutation tumors can be seen in any location in the CNS, over a third are located at midline. Given location, these tumors are less often biopsied and standard treatment with chemoradiation is often initiated blindly under the assumption that pediatric low-grade gliomas have similar prognoses [101]. However, these tumors confer a poor outcome with increased risk of progression and transformation, particularly when associated with cyclin dependent kinase inhibitor 2A (CDNK2A) [101, 102]. This has led to a great interest in selective *BRAF*^V600^ therapies.

The 2018 VE-BASKET study, a non-randomized multicohort analysis of *BRAF*^V600^-mutant gliomas, showed that vemurafenib, a selective *BRAF*^V600^ inhibitor, exhibited antitumor activity in some patients [103]. A follow-up study in 2021 by Berzero et al. showed long term clinical benefits to targeted therapy of *BRAF*^V600^-mutant brain tumors in adult patients [104].

Aside from primary CNS tumors, BRAF mutations have been implicated in several cancers including melanoma, pancreatic acinar carcinoma and papillary thyroid carcinoma [105]. In particular, 40–60% of melanomas can exhibit the mutation. Melanoma has been shown to metastasize to the brain in ~ 7% of all cases and up to 75% of those with stage IV disease regardless of whether the BRAF mutation is possessed [106–108].

It has been shown that primary melanoma tumors and their brain metastases do not always share the same mutational status [109]. This can have treatment altering consequences as intracranial melanoma metastases with *BRAF*^V600^ mutation can be targeted with the inhibitors dabrafenib and vemurafenib [110]. Anti-BRAF therapies for those tumors with BRAF fusions have been limited to date [105]. Not only may BRAF inhibitors be ineffective against BRAF fusion driven malignancies, but tumor progression and/or BRAF inhibitor resistance may be promoted [105, 106].

### BRAF mutant tumor imaging

There is considerable overlap in the imaging appearance of pleomorphic xanthoastrocytoma, ganglioglioma and pilocytic astrocytoma, which classically present as a cyst with enhancing nodule [111]. However, there has been ongoing research in discovering novel imaging manifestations to discern between these tumors as well as predict BRAF status.

**Fig. 6:**
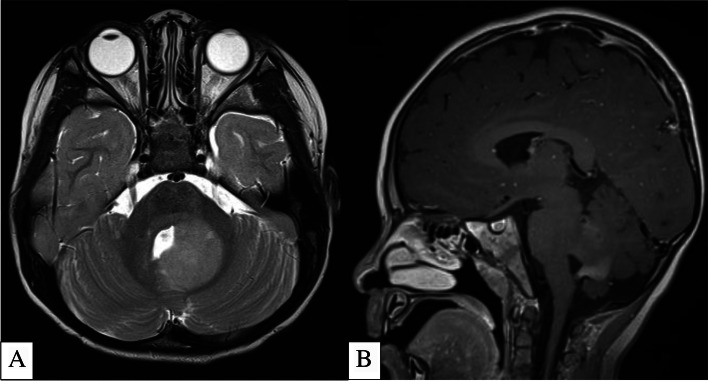
**A-B** 3-Year-Old Patient with Biopsy Proven Left Cerebellar Ganglioglioma. Axial T2-Weighted MRI (**A**) shows an expansile, infiltrative, homogenously T2 hyperintense lesion centered at the mesial aspect of the left cerebellar hemisphere and left middle/superior cerebellar peduncles. Sagittal T1-Weighted Post Contrast MRI (**B**) shows the classic “paintbrush” enhancement within the lesion

For example, Lindsey et al. showed that infratentorial (posterior fossa) gangliogliomas tended to be infiltrative and expansile with “paintbrush” enhancement (Fig. 6). In contrast, supratentorial gangliogliomas tended to be well circumscribed with heterogenous enhancement [112]. A recent study by Ramaglia et al. found that *BRAF*^V600^-mutant pilocytic astrocytoma and gangliogliomas had significantly lower ADC values compared to wildtype regardless of location and tumor histology [113].

While currently the only way to confirm the molecular landscape is by tissue sampling, there is growing feasibility and supportive evidence behind radiomics-based prediction of BRAF status. Wagner et al. also showed positive exploratory results when they applied radiomics and machine learning on FLAIR images of pediatric low-grade gliomas for the prediction of BRAF status [102]. Likewise, Shofty et al. showed a proof of concept for virtual biopsy using radiomics analysis for the non-invasive diagnosis of BRAF mutation status in those patients with intracranial melanoma metastases. Radiomic analysis of MRI exams from a small sample of 54 affected patients with known BRAF status was performed and subsequently submitted to machine learning. The results showed an accuracy, positive predictive value and negative predictive value of 78%, 81% and 75.8%, respectively. [106] Though the results lag that of traditional histology-based results, it may still prove useful in polymetastatic or poor surgical candidate patients [106].

## Conclusion

Currently, most CNS tumors require tissue sampling to discern their molecular/genomic landscape. However, growing research has shown the powerful role imaging can play in non-invasively and accurately detecting the molecular signature of these tumors (Table 1). Certainly, the burgeoning fields of AI/radiomics/radiogenomics have buoyed such research and contributed to considerable fanfare. It is important to note that none of the reported AI/radiomic/radiogenomic models have been validated prospectively and thus the clinical implementation remains speculative. Nonetheless, we believe that further supportive work in neuroimaging will have promising longitudinal consequences toward helping select patients who may benefit from novel therapies.

**Table 1:** Molecular markers, their relevant tumors and imaging features

Marker	Relevant Tumors with these Mutations	Relevant Imaging Features
IDH-1/2 Mutation + 1p19q Non-Codeletion	IDH-Mutant Astrocytoma	T2/FLAIR Mismatch [17–19]
IDH-1/2 Mutation + 1p19q Codeletion	Oligodendroglioma	Frontal Lobe Predominant, Poorly Defined, Heterogenous Mass with Calcifications [12–15]
EGFRvIII Mutation	Glioblastoma	Frontoparietal Predominance with Radiomic Signature of Higher rCBV, Lower ADC, Higher FA, and Lower T2-FLAIR Signal [38]
H3 K27-Altered	Pediatric Diffuse Midline Glioma	Variable Midline Brainstem Mass Often with CSF Dissemination [61–63, 73]
FGFR3-TACC3 Fusion	Adult-Type Gliomas	Non-Eloquent Area Involvement with Poorly Defined Margins and Reduced Enhancement Intensity [39]
TERT Promoter Mutation	Adult-Type Gliomas	Higher Volume of Necrosis and Lower Vascular Permeability Values [52, 53]
WNT	Medulloblastoma	Epicenter at the Cerebellar Peduncle [74, 75, 81, 82]
SHH		Adult-Epicenter at the Cerebellar Hemisphere Infant-Ill-Defined Margins and Prominent Enhancement [74, 75, 81, 82] Low to No Taurine or Creatine Levels [83, 84]
Group 3		Epicenter at Midline with Ill-Defined Margins and Prominent Enhancement [74, 75, 81, 82] Taurine Peaks and High Creatine [83, 84] Laminar Metastases [90]
Group 4		Epicenter at Midline with Well-Defined Margins and Trace to No Enhancement [74, 75, 81, 82] Taurine Peaks and High Creatine [83, 84] Nodular and/or Suprasellar Metastases [90]
BRAF^V600^ Mutation	PXA, PA, GG	Cyst with Enhancing Mural Nodule [111] *BRAF*^V600^-Mutant PA and GG Show Significantly Lower ADC Values [113] Supratentorial GG-Well Circumscribed with Heterogenous Enhancement [112] Infratentorial GG-Infiltrative and Expansile with “Paintbrush” Enhancement [112]
BRAF-K1AA1549 Fusion	PA	

## Data Availability

Not Applicable.

## Abbreviations

AI: Artificial Intelligence
CNS: Central Nervous System
IDH: Isocitrate Dehydrogenase
WHO: World Health Organization
EGFR: Epidermal Growth Factor Receptor
H3 K27-altered: Histone H3F3A
FGFR3-TACC3: Fibroblast Growth Factor Receptor 3-Transforming Acidic Coiled-Coil Containing Protein
TERT: Telomerase Reverse Transcriptase
PCV: Procarbazine, CCNU/Lomustine and Vinscristine
NAA: N-Acetylaspartate
FDG: 2-Deoxy-2-[18F]fluoro-D-glucose
MET: Methyl-11C-L-methionine
FDOPA: 3,4-Dihydroxy-6-[18F]-fluoro-L-phenylalanine
FET: 18F-fluoroethyltyrosine
2HG: 2-Hydroxyglutarate
CAR: Chimeric Antigen Receptor
rCBV: Relative Cerebral Blood Volume
FA: Fractional Anisotropy
DWI: Diffusion Weighted Imaging
ADC: Apparent Diffusion Coefficient
WNT: Wingless Type
SHH: Sonic Hedgehog
MAPK: Mitogen Activated Protein Kinase
PA: Pilocytic Astrocytoma
PXA: Pleomorphic Xanthoastrocytoma
GG: Ganglioglioma
MEK: Mitogen-Activated Protein Kinase Kinase
CDNK2A: Cyclin Dependent Kinase Inhibitor 2A
